# Applications of Telemedicine in Dermatology

**DOI:** 10.7759/cureus.27740

**Published:** 2022-08-07

**Authors:** Eshita Sud, Ashish Anjankar

**Affiliations:** 1 Department of Medicine, Jawaharlal Nehru Medical College, Datta Meghe Institute of Medical Sciences, Wardha, IND; 2 Department of Biochemistry, Jawaharlal Nehru Medical College, Datta Meghe Institute of Medical Sciences, Wardha, IND

**Keywords:** telehealth, digital dermatology, tele- dermatology, virtual care, telemedicine

## Abstract

Telemedicine is a technological tool that enhances well-being all around the globe. Practicing medicine or performing a clinical examination from a distance was a mere thought until this decade's pandemic hit the world. Telemedicine is practicing medicine sitting on one side of a globe and diagnosing and treating a different individual from the opposite part of the world. There is a long way to go for medical practitioners to execute an entire clinical examination analogous to an accurate clinical examination. Telemedicine is a supplement to a patient's total care, not a replacement for in-person doctor visits. Family doctors can easily access specialists using telemedicine, which enables them to monitor their patients closely. Numerous telemedicine systems, including store and forward, real-time and remote, or self-monitoring, are used worldwide for education, healthcare delivery and control, sickness screening, and disaster management. Even if telemedicine cannot solve every issue, it can significantly lessen the strain on the healthcare system. Nevertheless, investigations performed via telemedicine have started incorporating various medical instruments called telemedicine peripherals, including electronic stethoscopes, teleophthalmoscopes, and video-otoscopes. The prevailing disease around the globe of coronavirus has remarkably debilitated the medical infrastructure in providing diagnosis, treatment, monitoring, and follow-ups. As a result, there is a significant change in the way of practicing medicine and managing patients. Telemedicine provides timely patient care and reduces the risk of exposure to various communicable diseases offered to medical practitioners. The development of imaging technologies has significantly impacted dermatology, a specialty that relies on visual signals. Reviewing dermatology's existing situation and potential digital future, in brief, is the goal of this study. This study provides brief information on telemedicine, its application and scope in dermatology, and how it can alter the healthcare system.

## Introduction and background

In scientific words, telemedicine is "the usage of medicinal knowledge borrowed from one place to another via computerized transmission for improving the clinical health status of a patient." Telemedicine and telepath, as generally utilized today, can be considered interchangeable [1]. Broadcasting of radiologic photographs (teleradiology) is the most frequently employed and comprehensively researched telemedicine use. Another specialty of telemedicine that typically involves no patient contact is telepathology [2-4].

The oldest use of telecommunications in healthcare can be traced to the period of the Civil War when telegraphs were used for conveying casualty lists and ordering supplies [5]. The creation of the telephone, the radio, and other modern means of wireless and satellite-based communication further led to the advancement in medicinal telecommunication. The only documented reference to telemedicine comprises teleradiology and remote transmission and interpretation of radiographic images [6]. Improvements in technology have led to the growth and expansion of telehealth. As we entered the 21st century and with the advancement and development of the World Wide Web - which, through websites and its application, generated content from one user to another more accessible through a network that connected them across the globe despite having different electronic devices as the device on which they could work. Web 2.0 also aided in developing Voice Over Internet Protocol viable and other means of audiovisual communication. The mobile device and tablet-based industries have paid attention to the need for live discussions for medical practitioners to collaborate and debate more productively.

The COVID-19 epidemic has wreaked unmatched social and economic havoc with more than 37.8 million cases and over 1 million fatalities globally. The healthcare industry has seen a beneficial transition due to creative solutions that strive to lessen the harmful effects of COVID-19 on human health. For example, telehealth usage has increased due to the current public health crisis [7]. India, a developing and lower-middle-income nation, is now experiencing a lack of healthcare professionals, including physicians, nurses, and midwives. About 70% of Indians reside in isolated, rural communities without access to even the most basic medical services [8]. In such cases, telemedicine substantially contributes to providing quality and affordable healthcare to India's poorest citizens and is anticipated to close the health disparity between rural and urban areas. It is uncertain whether telemedicine technology will successfully offer adequate healthcare services to the underprivileged, isolated, and rural people [8]. Telehealth may, in the future, considerably improve health care, according to persuasive data. However, to fully utilize telehealth's potential and revolutionize healthcare for the entire world's population, its viability and implementation in resource-constrained environments and low- and middle-income countries must be established. A global agreement on definitions, limitations, protocols, oversight, evaluation, and data protection is urgently required, given the rapid development of telehealth [7].

## Review

Opinions of doctors and patients

It is an evident fact that telemedicine is an expanding field in the healthcare system. It is considered an innovative transformation within the healthcare industry. Regular patient communication, improved patient outcomes, more satisfactory treatment compliance, closing healthcare loopholes, more patient appointments per day, fewer no-shows and late cancellations, a comfortable and flexible lifestyle, higher satisfaction, and more engaged patients who are better able to compete with retail care are just a few benefits for doctors. As recent technologies are making telemedicine more comfortable and affordable, many medical practitioners realize the advantages of incorporating telemedicine into their routine. Most doctors believe enhancing the quality of care is their foremost reason for enforcing telemedicine, as it helps them deliver timely patient care. Many agree that most patients need not be seen in person to receive care. In a clinical context, many healthcare executives have less pressure, giving them more time to concentrate on patient care [9].

Many doctors are drawn to provide online and tech-savvy care, especially the younger generations entering the medical field. Benefits seen in case of patients are frequent communications with their physician, open access to specialists, saved time and expenditure on travel (especially for rural patients), contact with the medical practitioners at their convenience, reduced wait hours, convenient, on-demand care, motivate them to be actively engaged with treatment decisions. More than 50% of the patients are satisfied sharing their concerns with their physicians using various modes of telecommunicating. Statistics involving patients' perspectives toward telemedicine prove that 76% prioritize gaining access to healthcare over their need for face-to-face interaction with physicians [10]. If they had access to telemedicine services, about 16% of these people would choose to visit the emergency room for a mild illness. A study conducted among 8,000 patients said that there is no difference between virtual appointments and in-person hospital visits. More than two-thirds of patients say telemedicine gave them a sense of fulfillment with medical care [9].

Applications in dermatology

It is inexplicable how telemedicine is used in traditional healthcare, given the effectiveness of online consultations for healthcare. Puskin and Sanders (1995) [11] divided the number of impediments to the full implementation of telemedicine systems into three categories: (1) technological or organizational infrastructure of telecommunications; (2) organizational or human infrastructure of organizations; and (3) financial infrastructure of healthcare.

History

To create a picture that was 3 feet and 2-1/2 feet in size, Murphy et al. (1972) [12] projected a collection of 75 color images onto a projection screen. This study was the first analysis of dermatologic diagnosis via television. Doctors judged from direct observation of the slides or the transmitted visuals. The precision was inferior for the broadcast photos than for the falls compared to the known diagnosis, probably due to the techniques used. Color images produced incredibly accurate than black and white.

Some other studies on teledermatology in the past have been briefly described in Table 1.

**Table 1:** Various Studies Assessing Teledermatology for Education The table illustrates how various authors performed numerous studies to evaluate the future of teledermatology, describing the type of study conducted, their objectives, population taken into consideration, and conclusions derived [19].

Title	Authors	Study Type	Objective	Population	Conclusion
During a simulated in-training exam, dermatology residents compared ocular and glass slide microscopy.	Brick et al. [13]	Survey	Compare the opinions of dermatology residents toward traditional glass slide microscopy with virtual microscopy in terms of diagnostic accuracy.	Dermatology, fellows, and residents	Overall, glass slides had a higher diagnostic accuracy than virtual slides (P=0.01). Virtual microscopy did not generally receive a personal preference over glass slides.
During a simulated in-training exam, residents in pathology and dermatology were randomly assigned to either virtual microscopy or glass microscopy.	Berger et al. [14]	Randomized comparison	Determine whether residents' exam results would be comparable if administered using the two image forms; evaluate the respondents' perceptions of the two methods.	Dermatology residents and fellows	Despite their general preference for the latter, residents were equally adept at accurately diagnosing dermatopathology patients when assessed using virtual and glass slide microscopy.
Residents and medical students can learn about dermatology using teledermatology as a teaching method.	Boyers et al. [15]	Survey	Assessment of teledermatology's effectiveness in teaching the six essential clinical competencies and how dermatology residents and medical students perceive it.	Dermatology resident; Medical students	Tele-dermatology is a crucial educational tool, according to residents (79%) and medical students (88%) alike. In patient care, medical knowledge, practice-based learning, improvement, and systems-based practice, teledermatology is appreciated as a teaching tool for dermatology.
Diagnostic and treatment agreement between the resident and attending dermatologists using teledermatology as a teaching tool.	Nelson et al. [16]	Prospective study	Analyze the diagnostic and treatment agreement between the resident and attending dermatologists responding to primary care providers' stored and forwarded teledermatology consults.	Dermatology residents and attendings	Diagnoses and management plans between resident and attending dermatologists were fully concordant for 53% and 65% of dermatologic conditions, respectively. Data collected reflected that there is some lack of agreement regarding diagnosis and management plans among attending and resident dermatologists for conditions observed in dermatology as 47% and 35%, respectively.
Dermatology-Specific Digital Education for Health Professionals: A Systematic Review by the Digital Health Education Collaboration.	Xu et al. [17]	Cochrane review approach	Analyze the data to determine whether digital dermatology education helps enhance knowledge, skills, attitudes, and satisfaction.	Health professionals	The primary learning outcomes were comparable regarding information gain, skill development, and satisfaction, indicating the potential for using digital health education as a supplement or replacement for conventional dermatology education.
Education in Tele-Dermatology: Use of Tele-Dermatology in US Residency Programs.	Wanat et al. [18]	Descriptive survey	To learn about dermatology training schools' present use of telemedicine and any future interest in it.	Dermatology residency programs	Out of the 72 respondents, 34 (47%) programs used telemedicine as a component of their residency training, with store and forward technology being the most popular (85%) and live interactive (35%) or a combination of the two techniques being used. The survey results reveal a training gap for dermatology residents in telemedicine.

Teledermatology, in particular, took part in that development and is now a potent instrument in dermatological consultation because of the visual nature of the dermatological specialty [20]. Teledermatology could help identify and prevent occupational skin problems early on. Contemporary smartphone apps may facilitate using self-monitoring tools by employees in high-risk professions with artificial intelligence technologies [21].

Store and forward technology, real-time interactive technology, or a hybrid method incorporating aspects can all be used for teledermatology. Teledermatology is a reliable diagnostic method for identifying skin problems. The evidence for accurate diagnosis has been less clear. Store and forward teledermatology and real-time interactive teledermatology result in average reductions of 45.5 percent to 61.5 percent in in-person dermatological consultations. Clinical outcomes for patients who receive teledermatology care or are managed by it are on par with traditional medicine. Overall, teledermatology is well-liked by patients. Accessibility and reduced travel time are mentioned as advantages [22].

Through teledermatology, teledermatologists can remotely analyze diagnostic photos of skin problems with associated clinical histories using a variety of modalities, such as photographic clinical images or live video teleconferencing. Then, diagnoses and suggested therapy courses can be provided and carried out remotely. The data support the accuracy and cost-effectiveness of its diagnosis and treatment. This dynamic field's administrative, regulatory, privacy, and reimbursement policies are constantly changing [23]. A potential technique for diagnosing and treating ambiguous pigmented skin lesions is teledermoscopy. Mobile teledermatology and mobile teledermoscopy have recently been shown to be feasible, and they have the potential to develop into universally accessible tools. They might pave the way for a novel adaptable triage approach to identify melanoma and skin cancer in general [24].

In practically every area of dermatology, including research, clinical practice, dermatology education and training, and the prevention, diagnosis, and treatment of diseases as well as patient follow-up, digital dermatology has found application. Smartphone programs like VisualDx, MyDermPath, and YouDermoscopy function as diagnostic aids and can improve a user's dermatology expertise. Tools like multispectral digital skin lesion analysis (MSDSLA) increase the accuracy of diagnoses and reduce the need for pointless biopsies. Due to reduced wait times and lower expenses, teledermatology promotes patient satisfaction. Rural areas and underserved groups are more prone to receive a dermatologic assessment via teledermatology [19]. One can undertake teledermatology consultations utilizing real-time or store-and-forward technologies. Dermatologists employing store-and-forward and real-time modalities can attain good diagnostic reliability comparable to clinic-based examiners. Regarding diagnostic accuracy, there is less evidence available. According to recent research, teledermatologists analyzing store-and-forward consults are as accurate as dermatologists who work in offices. About 25% of in-person clinic appointments are avoided when store-and-forward consult technologies are employed. A real-time consult technology eliminates the requirement for nearly two out of every three clinic visits. When compared to a traditional referral procedure, store-and-forward technology allows for quicker treatments for patients [22]. Initially focusing on skin cancer, notably melanoma, AI research in dermatology has expanded more recently to include various diagnoses and treatment suggestions [25].

Teledermatology provides specialized medical care with reduced diagnostic precision and a more significant proportion of incorrect diagnoses. As a result, given that it is a new technique, it should be used with extreme caution concerning illnesses with lower diagnostic accuracy [26], such as inflammatory dermatoses and skin cancers, has been indicated. Teledermatology must defend the patient's right to autonomy, professional privacy, confidentiality, and intimacy. The clinical history must reflect the patient's implicit will and at least verbal consent, if appropriate, from the patient, a responsible family member, or the patient's legal guardian. The doctor must have direct knowledge of the patient's clinical history or have access to it during medical care to safeguard the patient's privacy and intimacy and the doctor-patient relationship. The method of telecare used to conduct the consultation, the suggestions, and the recommended medical care should all be written in the patient's medical file [26]. An information method on the goals and uses of the images captured and sent is necessary for the practice of teledermatology. Any teledermatological application must adhere to the regulations' criteria for confidentiality and privacy protection of personal data [27]. Additionally, concerning teledermatology, medical practitioners must take extra care to ensure that the images are encrypted or coded when they are updated or added to the clinical history to avoid access by unauthorized individuals [28].

Patient Protection and Affordable Care Act (ACA)

To promote the use of telemedicine, "The Patient Protection and Affordable Care Act (ACA) comprises four divisions, including the establishment of the Center for Medicare and Medicaid Innovation (CMMI) within the Centers for Medicare and Medicaid Services." Focusing on these aspects will ultimately facilitate the usage and elaboration of telemedicine. The ACA includes several services to support telemedicine and gives the new CMMI the ability to research and develop innovative care models that adopt technology, such as electronic monitoring, in various care settings. The ACA states that CMMI is also in charge of researching the utilization of organizations in medically underserved areas and the facilities used by Indian Health Services. The project looks at the provision of telemedicine services in managing behavioral health disorders and stroke, in addition to initiatives to increase the ability of doctors and other non-medical individuals to deliver health care for people with long-term and complex medical diseases.

Openings for telehealth applications for Medicare beneficiaries form a part of the ACA. Additionally, the law needs accountable care organizations (ACOs) to develop strategies to advocate evidence-based medicine, patient involvement, records standards, and cost estimates and coordinate care using telemedicine, distant patient monitoring, and other supporting technologies. The Independence at Home Demonstration Program created by this law allows distance observation that the policy describes as "home-based primary care providers" (i.e., a team of doctors and nurses offering health care facilities in homes of patients having various chronic ailments) [6].

Telemedicine and India

Given that over 121 crore diverse people are living in India, unbiased organization of medical services has emerged as a critical goal in public health management. Moreover, the healthcare industry's current trend is concentrated in cities and towns far from rural India, where 68.84% of India's population resides [29]. A Telemedicine Pilot Project was initiated by the "Indian Space Research Organization (ISRO) in 2001 to link the Apollo Hospital in Chennai with the Apollo Rural Hospital in Aragonda, Andhra Pradesh's Chittoor district" [30]. For the sake of increase in the availability of telemedicine services in India, "ISRO collaborated with the Department of Information Technology (DIT), the Ministry of External Affairs, the Ministry of Health and Family Welfare, and the state governments."

"The Ministry of Health under the Government of India has included initiatives like the Integrated Disease Surveillance Project (IDSP), National Cancer Network (ONET), National Rural Telemedicine Network, National Medical College Network, and the Digital Medical Library Network" to consolidate the public health data that is currently available and provide easy entry. Two other praiseworthy initiatives included the organization of a "National Telemedicine Task Force by the Health Ministry in 2005 and the creation of standardized telemedicine practice guidelines by the Department of Information Technology in the Government of India". "International assignments like the Pan-African network Project and the SAARC (South Asian Association for Regional Co-operation) Telemedicine Network Projects" have also been accepted as an endeavor of the External Affairs Ministry, strategically positioning Indian telemedicine in the international system [31,32]. Some effectively appointed telehealth services in India include mammography services at "Sri Ganga Ram Hospital, Delhi; oncology at the Regional Cancer Center, Trivandrum [33]; surgical services at Sanjay Gandhi Postgraduate Institute of Medical Sciences, School of Telemedicine and Biomedical Informatics". When medical care becomes urgently necessary, telemedicine is also used in areas where huge people occasionally or regularly congregate. For instance, the Government of Uttar Pradesh utilizes telemedicine at the time of Maha Kumbhamelas.

One field that has been successful at piquing the commercial sector's attention and encouraging them to participate actively in public health management is telemedicine. "Narayana Hrudayalaya, Apollo Telemedicine Enterprises, Asia Heart Foundation, Escorts Heart Institute, Amrita Institute of Medical Sciences, and Aravind Eye Care" are a few of the country's most significant private sector telemedicine operators at the moment. Governments at the federal and state levels and organizations like ISRO support them in their operations by providing them with the latest and most relevant technology [34]. The ISRO telemedicine network has advanced significantly over the past few years. It has grown to connect 15 super-specialized and 45 remote and rural hospitals. The Andaman and Nicobar and Lakshadweep islands, the mountainous areas of Jammu and Kashmir, the Medical College hospitals in Orissa, and a few of the rural/district hospitals in other states are among the remote nodes [35].

India currently only has a doctor-to-patient ratio of 0.62:1000, much below the WHO's recommended ratio of 1:1000 [36]. Since it costs money to train new doctors, the doctor-to-patient balance is anticipated to remain reduced for a very long period (time and money). Partially addressing this gap are the numerous regions in the country that have active telemedicine services. "The Ministry of Health and Family Welfare and the Department of Information Technology" jointly oversee telemedicine services nationwide. The "National Rural Telemedicine Network for e-Healthcare delivery and the National Medical College Network (NMCN)" for interconnecting medical colleges across the nation have been established by the telemedicine division of the Ministry of Health and Family Welfare (MoHFW), Government of India [37].

Village Resource Center (VRC)

ISRO created the VRC concept to offer various services, including weather forecasting, tele-education, telemedicine, online decision support, interactive farmers' advisory services, tele-fishery, and e-governance. The VRCs serve as learning facilities and connecting points to specialty hospitals, offering specialized medical care to the villages. Almost 500 such VRCs have been constructed [38].

Future of telemedicine

**Figure 1:**
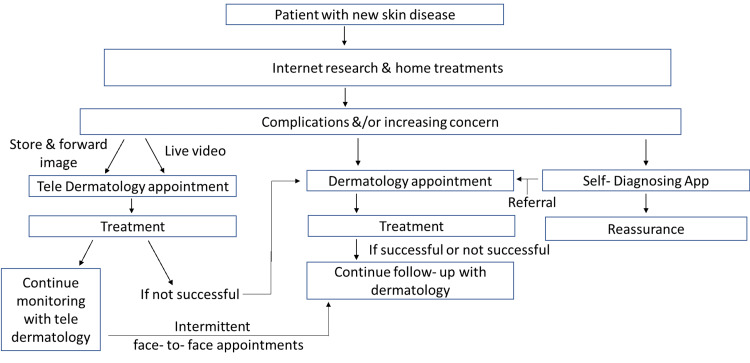
Digital Future of Teledermatology The given figure describes how the clinical practice of dermatology will change in the future. It explains how patients with new skin diseases will reach a doctor via teledermatology appointment, a physical appointment with a dermatologist or a self-diagnosing app and, eventually, how the case will be resolved [19].

The aim and vision of incorporating telemedicine and virtual care in the healthcare system were to deliver optimum healthcare, which facilitated addressing long waiting hours and the threat of disease progression in social distancing, aid hospitals, and clinics. Reduction in real-time visits to the medical centers and minimizing one-on-one interchange between physicians and their patients, virtual treatment, and monitoring solutions decrease the transmission of potent microbiological agents and shield medical practitioners from diseases. Telemedicine and telehealth have also successfully managed serious acute respiratory infections like Severe Acute Respiratory Syndrome (SARS) and Middle East Respiratory Syndrome (MERS). With the advent of COVID-19, canceling and postponing many in-person outpatient medical appointments have been prevalent. A flowchart on the future of teledermatology has been described in Figure 1.

Some of the problems one might face with telemedicine will be technical, mainly associated with internet access, Wi-Fi strength, and bandwidth issues [39-41]. Hence, occasionally medical practitioners favor voice calls for consultation over videoconferencing modalities as lacked stable connections. Similarly, it is a need of the hour to have software that can translate real-time data for medical and paramedical staff and the patients so that there are no language barriers.

## Conclusions

Telemedicine and online applications or websites empower the medical fraternity by providing aid in managing extensive outbreaks and emergencies in highly unpredictable times. Technology in terms of telecommunication in medicine has been used in the past and does not belong to the discoveries of the 21st century. One of its uses was connecting with remote regions and providing adequate treatment to patients with a good prognosis. Telehealth is extensively available, affordable, and accepted by medical practitioners; with COVID-19 spreading and various cities undergoing lockdown due to the high infectivity of the coronavirus, the use of telemedicine by medical practitioners as a tool of diagnosis and treatment was considered an obligation. The usage of telecommunication in the world of medicine gives access to safe, affordable, and appropriate care. Nevertheless, it has a long way to go owing to various problems like the unavailability of required tools, insufficient funds, and lack of expertise.
